# Simultaneous bilateral rupture of patellar tendons in diabetic hemodialysis patient: A case report

**DOI:** 10.22088/cjim.9.3.306

**Published:** 2018

**Authors:** Ali Torkaman, Alireza Yousof Gomrokchi, Omid Elahifar, Pooyan Barmayoon, Seyedeh Fahimeh Shojaei

**Affiliations:** 1 Bone and Joint Reconstruction Research Center, Shafa Orthopedic Hospital, Iran University of Medical Sciences, Tehran, Iran; 2 Firoozgar Clinical Research and Development Center, (FCRDC) , Iran University of Medical Sciences, (IUMS) , Tehran, Iran

**Keywords:** Bilateral, Hemodialysis, Patellar tendon, Rupture

## Abstract

**Background:**

Bilateral rupture of the patellar tendon is a very rare injury, which takes place in relation to chronic systemic diseases. These injuries are known causes. Some of these causes are particular in patellar tendon rupture and another are in quadriceps tendon rupture.

**Case presentation:**

70-year-old diabetic man with simultaneous bilateral patellar tendon disruption of proximal insertion without trauma, receiving long-term hemodialysis.

**Conclusions:**

In the present study, we report a case of patellar tendon rupture that has two differences with literature: first, renal failure is a known risk factor for quadriceps tendon rupture, and secondly, the prevalent age of patellar tendon rupture is less than 40 years. Clinical picture, diagnosis, pathogenesis and treatment are discussed. Finally, the literature is reviewed based on previous studies.

The disruption of the extensor mechanism of the knee is commonly caused by the fracture of the patella in most cases. With regard to other causes, we can refer to the disruption of the quadriceps mechanism and disruption of the patellar tendon. Further, patellar tendon rupture or avulsion is more common in patients younger than 40 years old, especially athletes. Quadriceps rupture is more common in older patients and in patients with systemic disease or degenerative changes. Systemic diseases such as lupus erythematous, diabetes, gout, hyperparathyroidism, uremia, and obesity are related to the disruption of the quadriceps mechanism. Furthermore, a relationship among prior steroid injection, the use of corticosteroids or fluoroquinolone antibiotics, and tendon rupture has been documented in some studies (1). Patellar tendon rupture is a rare injury (2), which is regarded as the third cause of disorder in the knee extensor mechanism (3), irrespective of patella fracture and quadriceps tendon rupture. Further, patellar tendon rupture, which ranked 1 to 43, among these three common injuries (4), is regarded as a disabling injury (5) that happens sporadically among athletes during matches, hard practices, running, or jumping(6) in their 30s or 40s (7). Bilateral rupture of the patellar tendon is extremely rare and has been reported in the form of case report so far (8). Some factors play a pivotal role in making the individual more susceptible, such as rheumatoid arthritis (8), systemic lupus erythematous (9), arteriosclerosis (9), mellitus diabetes (10), hyperparathyroidism (11) and renal failure (12). In this study, we report a case of 70-year-old diabetic man with simultaneous bilateral rupture of patellar tendon. Given that the patient was elderly, it was assumed that he was diagnosed with quadriceps tendon rupture. However, his both patellar tendons were torn simultaneously, which rarely takes places in such cases. Regarding the existing case, we highlighted the possibility of tearing patellar tendon rupture in elderly people by taking the diagnosis into consideration.

## Case Presentation

After explaining the condition and the type of this study, we gained the consent of the patient about reporting his situation. This study was presented before the Ethics Committee of Iran University of Medical Sciences. A 70-year-old man with 80 kg weight with symptoms such as sharp pain in knees, swelling, bruising, and sensitivity to touch called the "emergency ward" and was considered for the purpose of this study. He was not able to stand on his feet and carry his weight because of falling down while getting off the car. Studying the background information, he was diagnosed with diabetes .He had been under diabetic care for five years due to nephropathy and renal complications.

Based on physical examinations, he was suffering from swelling in both knees and was unable to do active knees extension and weight-bearing tasks. Further, there was a gap under both patella in deep touch. His neurovascular examination was normal and he was not experiencing any problems or complaints in other limbs. Also, the man was hospitalized in the orthopedic ward and radiography was performed on him. 

**Figure 1:**
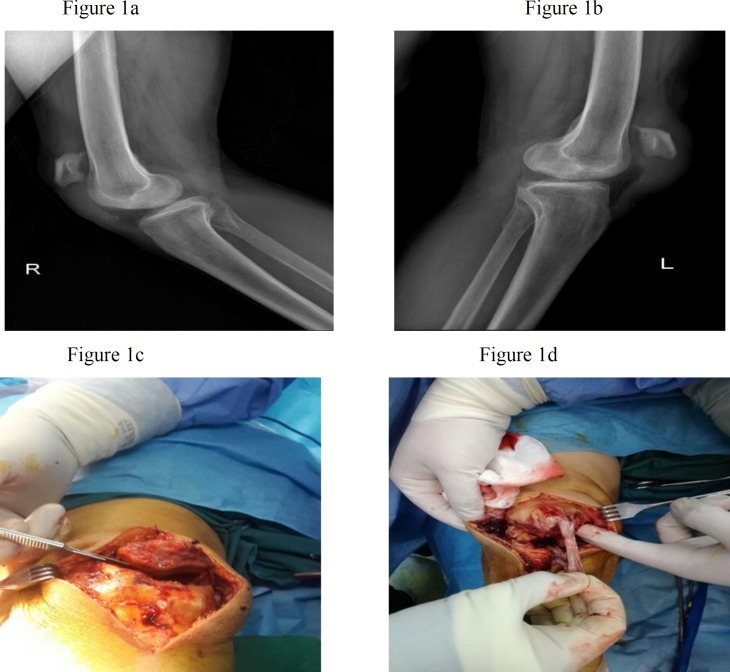
Rt and Lt lateral radiography of knee shows patella Alta, (a) and (b). Intraoperative photography show complete patellar tendon rupture from proximal attachment, (c) and (d)

**Figure 2:**
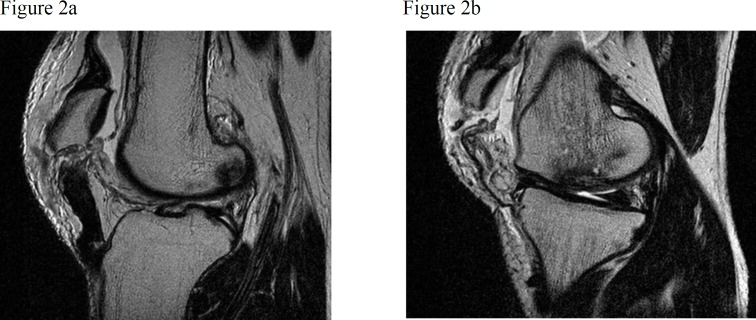
(a) MRI of bilateral rupture of patellar tendons in left knee (b) MRI of bilateral rupture of patellar tendons in right knee.

In a lateral knee x-ray, there was bilateral patella alta (figure1), although it was not confirmed in other types of radiography. Based on the MRI, the diagnosis of bilateral rupture of patellar tendons and the level of tendon rupture were confirmed (figure 2). Bilateral patellar tendon repair was performed for the patient within 10 days after the accident. Spinal anesthesia was administered after the patient was transported to the surgery room. He was transferred onto the operating table in the supine position and one gram of cefazolin was infused intravenously. Tourniquets were closed for both limbs. Both lower extremities, from the toe tip to the groin, underwent skin preparation and sterile draping in a sterile condition.

**Figure 3:**
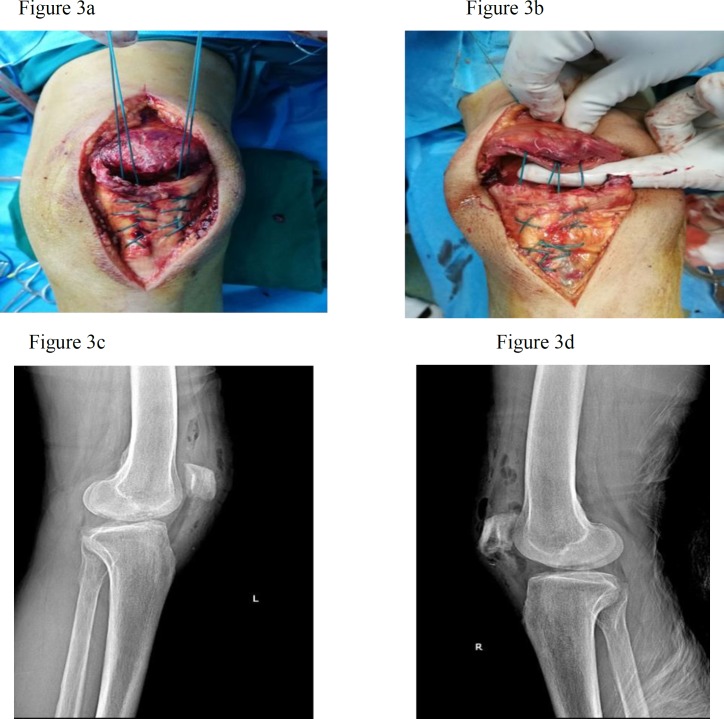
Technique of patellar tendon repair with bone tunnel, (a) and (b). Post-operative lateral radiography of Lt and Rt knees showing patella in normal position, (c) and (d)

**Figure 4:**
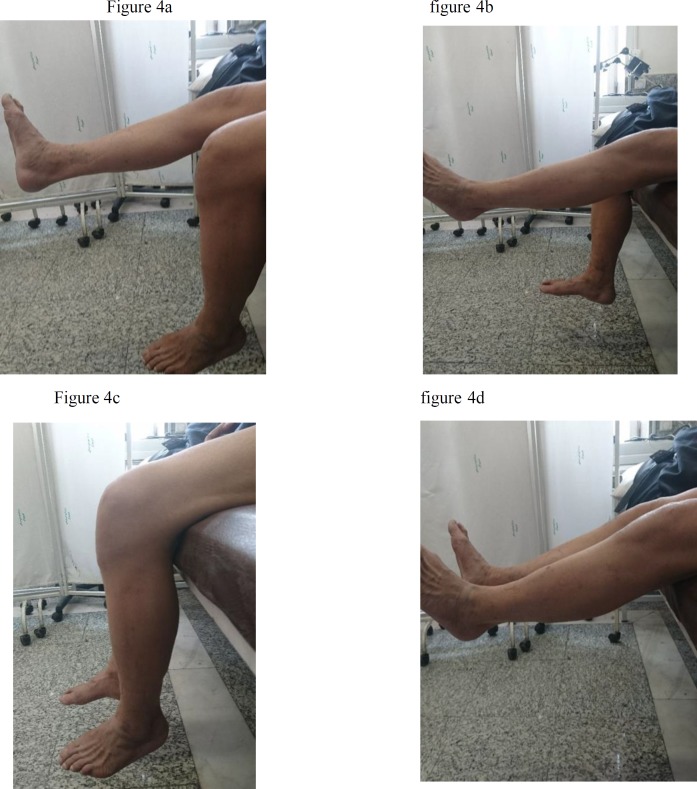
Range of motion of right and left knee (a,b, c, d)

First, the tourniquet on the left lower extremity was activated under pressure of 350 mm Hganda 15cm long incision was made in the anterior knee. After removing subcutaneous tissues, the quadriceps tendon and patella tendon were exposed. They were completely torn apart from distal pole of patella, which was extended to the medial retinaculum. Then, the patella tendon was sewn by using a continuous lock suture technique. Four strings were passed through three channels, created along the patella. After freshening the distal pole and creating a trough in the area, the strings were pulled taut and tied. Also, the retinaculum was sewn. After the completion of the repair, there was no need to place a circumferential tension suture of No. 5 as non-absorbable box wire. Subcutaneous and cutaneous tissues were sutured in separated layers and the tourniquet was inactivated. By implementing the method, other knees, troubled with the same complete rupture of the patella tendon from distal pole were repaired, along with retinaculum rupture (Figure 3). No sign of fracture was observed in the bones of both knees. Finally, a knee immobilizer was used for both lower limbs. Crutches were to be used for ambulation in the knee immobilizer until it gains sufficient strength, which is usually realized about one week after surgery. The patient started physical therapy, known as "physiotherapy", six weeks after the surgery and light activities were done using a walker. It should be emphasized that motion gradually increased 10-15 degrees each week. Three months after surgical repair, range of motion of both knees increased to 0°/90° near to normal and the patient can walk full weight without using axillary crutches (figure 4).

## Discussion

The patient, due to the absence of trauma, was diagnosed with bilateral and simultaneous rupture of patellar tendons and he was suffering from diabetes and was under diabetic care, thanks to the nephropathy. According to the sources available, the rupture of the patellar tendon takes place in people under the age of 40, while bilateral rupture occurs very rarely among them. As for the patient considered for the present study, it was expected that the quadriceps tendon could be affected by the trauma as it often happens in people with more than 40 years of age and is accompanied by degenerative changes in the tendon (13). Patients older than 40 years of age are susceptible to extensor mechanism rupture of the knee in the level of quadriceps tendon in the superior patellar pole (5, 14-16). Nevertheless, the reported case is rare concerning a 70 year-old man with bilateral rupture of the patellar tendon, which is inconsistent with the foregoing studies. 

A force of 17.5 times the body weight was necessary for ruptureof the patellar tendon. Nonetheless, rupture of the tendon takes place with a very trivial force, due to fragility of the tendon or the link of the tendon to the bone junction (3, 11, 17, 18). Diabetes mellitus is regarded as a popular reason for a wide range of muscular disorders, including tenosynovitis, joint stiffness, and tendon contracture (19). Diabetic patients are often encountered with the problem in their knee functions. The involvement of quadriceps and patellar tendons has been observed in diabetic patients (20). It was observed that as compared to non-diabetic patients, function of tendons in diabetic patient is reduced by 13% (21). Diabetes decreases collagen content and (22), which, in turn, leads to the unusual structure of collagens and the fragility of tendons (23).

On the other hand, due to the developments in control and treatment of diabetes, the lifespan of the patient has considerably increased. Nephropathy is one of the complications of having diabetes for a long time leading to chronic kidney disease (CKD). CKD is one of the main reasons for performing dialysis in the cases of males. Tendon rupture is considered as one of the rare complications in patients under diabetic care (24). Also, there is an increase in the age of morbidity of patellar tendon rupture in patients with renal disorders (25). Moreover, some believe that no successive dialysis is accompanied by malnutrition in the long run, which is regarded as the main factor for tendon rupture (24, 26). Toxic accumulation of urea is the cause of fragility and rupture of tendons (27). As well, the mechanism of spontaneous tendon rupture in uremic patients with secondary hyperparathyroidism has been described as significant osteolytic bone resorption with osteoclasts at the site of tendon insertion in which previous studies have also shown to be related to higher levels of PTH, phosphate, and calcium. (25, 28-30)

Based on the age of the patient, having diabetes and being under the care of dialysis, diagnosis of the rupture of the patellar tendon was below our expectations and was, in fact, an exception. Due to scarcity of this type of tendon rupture, and close examinations not being possible for the extension of the knee and touching around the tendon, the diagnosis of these ruptures are challenged and may lead physicians to a failure in their diagnosis. Besides, delay in diagnosis results in a change in the walking pattern of the patient, so the patient walks with small steps and the injured leg swings out and thrown forward during the walk. Thus, instability is created in the knee, and the patient faces some difficulty in climbing the stairs (5).

Finally, more than 6 weeks of delay in performing the patella tendon surgery may pave the way for prolonging recovery or complications.

In conclusion based on the results, rupture of the patellar tendon requires a huge amount of force, the investigation of disease background and the consumption of the drug, it is suggested that the underlying factors play more significant roles than the age of the patient. Rupture of the patellar tendon may take place with trivial trauma in elderly people with disease backgrounds like diabetes and advanced kidney diseases, which should be pointed out at the time of diagnosis. More research is recommended for future cases and analytical studies.

## Conflict of Interest:

None
